# Breastfeeding and the developmental origins of mucosal immunity: how human milk shapes the innate and adaptive mucosal immune systems

**DOI:** 10.1097/MOG.0000000000000778

**Published:** 2021-09-22

**Authors:** Bassel Dawod, Jean S. Marshall, Meghan B. Azad

**Affiliations:** a Department of Pathology; b Department of Microbiology and Immunology, Dalhousie University, Halifax, Nova Scotia; c Manitoba Interdisciplinary Lactation Centre (MILC), Children's Hospital Research Institute of Manitoba; d Department of Pediatrics and Child Health; e Department of Immunology, University of Manitoba, Winnipeg, Manitoba, Canada

**Keywords:** breastfeeding, developmental origins of health and disease, human milk, microbiota, mucosal immunity

## Abstract

**Purpose of review:**

Breastfeeding provides passive immunity while the neonatal immune system matures, and may also protect against chronic immune-mediated conditions long after weaning. This review summarizes current knowledge and new discoveries about human milk and mucosal immunity.

**Recent findings:**

New data suggest that certain microbes in maternal milk may seed and shape the infant gut microbiota, which play a key role in regulating gut barrier integrity and training the developing immune system. Human milk oligosaccharides, best known for their prebiotic functions, have now been shown to directly modulate gene expression in mast and goblet cells in the gastrointestinal tract. Epidemiologic data show a reduced risk of peanut sensitization among infants breastfed by peanut-consuming mothers, suggesting a role for milk-borne food antigens in tolerance development. Cross-fostering experiments in mice suggest the soluble Toll-like receptor 2, found in human milk, may be critical in this process. Finally, interest in human milk antibodies surged during the pandemic with the identification of neutralizing severe acute respiratory syndrome coronavirus 2 antibodies in maternal milk following both natural infection and vaccination.

**Summary:**

Human milk provides critical immune protection and stimulation to breastfed infants. Understanding the underlying mechanisms could identify new therapeutic targets and strategies for disease prevention across the lifespan.

## INTRODUCTION

Mucosal membranes are composed of specialized epithelial cells covering a layer of connective tissue that lines the respiratory, digestive, and urinary tracts. The immune system, with its innate and adaptive components, is an essential element of the mucosa, which serves as the body's first line of defense against pathogens and the first point of contact for establishing tolerance toward innocuous antigens such as food proteins and commensal bacteria [1]. After full gestation, all of the basic mucosal immune elements are in place, but they exist in an immature state characterized by incomplete barrier integrity, deficiency in secretory immunoglobulin A (sIgA), reduced complement activation, and low levels of memory immune cells [2]. Therefore, infants depend on their mother's milk for passive immunity, and rely on milk and other exogenous factors to stimulate mucosal immune system development.

Besides essential nutrients, human milk is rich in cytokines, immunoglobulins, growth factors, microbiota, soluble receptors, immune cells, enzymes, lipids, and oligosaccharides [3^▪^,4^▪^,^5^]. These components are dynamic and change over time to match the needs of infants throughout lactation. Some factors also have a diurnal rhythm or fluctuate during a single feeding [6^▪▪^]. By the end of pregnancy, the mammary gland starts to produce colostrum, which is especially rich in bioactive factors that provide passive immunity to the neonate [7]. Transitional milk is secreted for about two weeks, followed by mature milk that comprises a thinner ‘fore-milk’ that becomes fattier toward the end of the feed, referred to as ‘hind-milk’ [8]. Milk composition is highly variable between women due to several fixed and modifiable factors, including genetics, diet, health status, environmental exposures, infant gestational age, and infant sex [6^▪▪^,^8^].

It is well established that breastfeeding is protective against infections and immune-mediated diseases, both during and beyond the lactation period [9,10]. For example, recent systematic reviews and meta-analyses have found that breastfed (vs. formula fed) infants have a 31% lower incidence of diarrhea and a 52% lower risk of mortality due to infectious disease in the first 2 years of life [10]. These effects are particularly critical in low resource settings where the burden of infectious disease is high and the access to clean water for preparing formula is low. In high resource settings, breastfeeding remains important for the prevention of chronic immune-mediated diseases such as asthma (9% reduced risk) and allergic rhinitis (21% reduced risk) [10]. Collectively, this evidence suggests a critical role for human milk in developing the mucosal immune system during early life, which will be the focus of this review. 

**Box 1:**
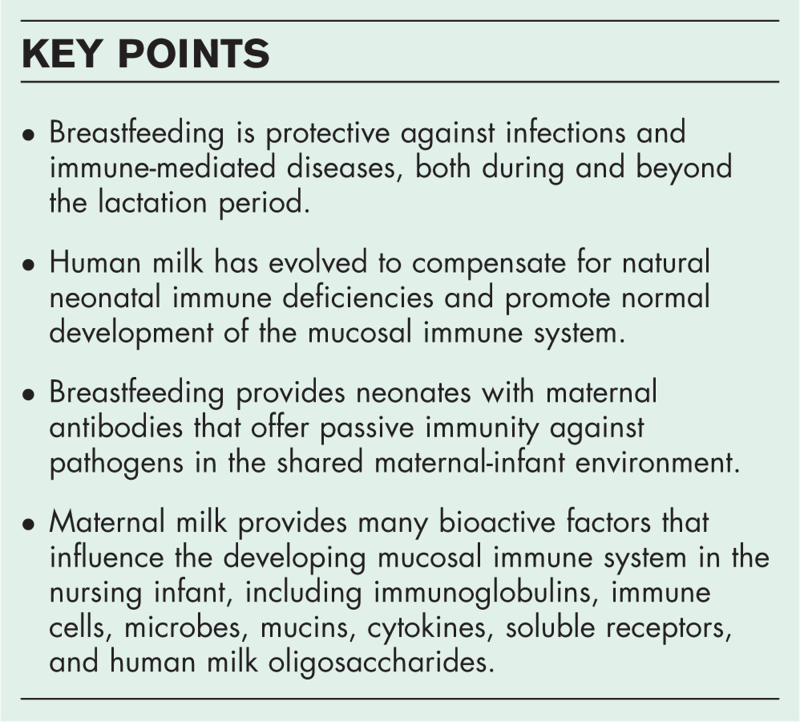
no caption available

## DEVELOPMENT OF THE MUCOSAL IMMUNE SYSTEM

**FIGURE 1:**
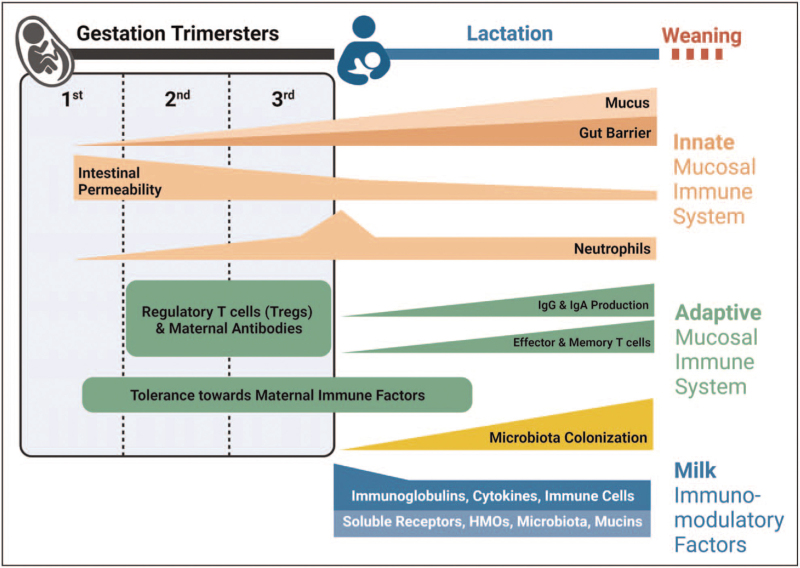
Timeline of innate and adaptive mucosal immune development during gestation and infancy and the bridging role of human milk. (Original). Gut barrier development beings *in utero* and continues until weaning, correlating with a decline in gut permeability and an increased thickness of the mucus layer. Levels of neutrophils increase toward the end of gestation and rapidly decrease to normal levels within the first hours after birth. Levels of Tregs are higher in the fetal period, which leads to immune suppression and tolerance toward maternal immune factors, such as immunoglobulins. After birth, the neonatal immune system gradually switches to an effector immune response with the increased exposure to environmental and milk immune factors, including microbiota. The innate and adaptive immune systems mature after birth, increasing the local production of mucosal antibodies. Milk components, such as microbiota, maternal cells, antibodies, cytokines, growth factors, soluble receptors, and HMOs, play an important role in promoting the normal development of the neonatal mucosal immune system (Figure 1); many are especially abundant in colostrum.

The mucosal immune system includes physical (epithelium) and chemical (mucus) barriers, signaling molecules (e.g. pattern recognition receptors), along with innate immune cells (neutrophils, macrophages, mast cells, natural killer T cells, dendritic cells [DCs]) and adaptive immune cells (B and T cells). These components overlay gastrointestinal, pulmonary, and other mucosal surfaces [11,12]. Structures of the fetal mucosal immune system are fully developed *in utero* by week 28; thus, neonates have a responsive but ‘immature’ immune system at birth [13]. With limited exposure to foreign antigens, the initial immune responses of neonates are essential for facilitating tolerance toward self, maternal, and bacterial antigens. However, they also have reduced functional capacity in some aspects of innate immunity. Thus, during this early developmental period, breastmilk is vital in helping to prevent infection and supporting a smooth transition from fetal to postnatal life as the mucosal immune system matures (Fig. 1).

### The innate mucosal immune system after birth

Innate mucosal immunity is the first line of defense against foreign antigens. It is less effective in neonates than adults, thus increasing the risk of infection in early life [14,15]. The gastrointestinal barrier and its gut-associated lymphoid tissue start to develop by 8–10 weeks of gestation [16] and continue after birth until weaning [17]. Increased intestinal epithelial permeability in the neonate allows passage of macromolecules, including maternal IgGs [18^▪^]. Intestinal permeability gradually declines after birth, and this process occurs more slowly in formula-fed infants [18^▪^,^19^,^20^], suggesting an essential role for breastmilk in such maturation. The mucosal epithelium is coated with a layer of mucus produced by goblet cells present at birth. Neonatal expression of certain mucins is low, resulting in reduced mucus layer thickness compared to adults [2].

Neutrophils are present in the fetus and increase in numbers before birth, declining to ’normal’ adult levels within few days of delivery [21]. However, their response to bacterial products is limited compared to adult neutrophils [15]. Monocytes and macrophages are also immature in neonates, with relatively low expression of innate receptors, such as Toll-like receptor 4 (TLR4) [22–24]. Moreover, the responsiveness of neonatal myeloid and plasmacytoid DCs to viral infections is limited, leading to attenuated priming of T cells and low secretion of interferons [25^▪▪^,^26^–^28^]. In addition, neonatal natural killer T cells, which are potent antiviral innate immune cells, produce limited interferon-γ and exhibit low cytolytic activity compared to adults [29].

The immaturity of the fetal/neonatal immune system provides opportunities for developing immune tolerance toward maternal antigens delivered *in utero* and via breastmilk, and for allowing optimal colonization by commensal microbes. During this period of vulnerability to infection, the infant relies on exogenous protection from maternal milk while its endogenous mucosal immune system matures.

### The adaptive mucosal immune system after birth

Humoral adaptive immune responses are mediated by antibodies produced by B cells, whereas cell-mediated responses are controlled by antigen-specific T cells. These adaptive immune responses are highly specific and long-lasting, generating immunological ‘memory’ that acts quickly to eliminate subsequent insults. Sensing of antigens by the innate immune response is the critical first step to developing adaptive immunity; thus, the immature state of innate mucosal immunity at birth leads to similarly immature adaptive humoral and cellular responses.

Functional B and T cells, the key elements of adaptive immunity, can be found as early as 12 weeks after birth in the gastrointestinal tract. However, these neonatal lymphocytes differ from mature adult lymphocytes, and their primary function is biased toward immune tolerance [30]. In addition, the high expression of transforming growth factor-β (TGF-β) in fetal mucosal lymphoid tissues [31,32] favors the expansion of regulatory T cells (Tregs) [33], which actively suppress B and T cells [34], resulting in low B-cell production of sIgA among neonates [35]. IgM-producing B cells predominate for the first month after birth before switching to IgA production, which keeps increasing until 2 years of age [36,37]. Neonatal secretion of sIgA at mucosal surfaces begins between 1 and 8 weeks of age [35].

## MATERNAL MILK AND MUCOSAL IMMUNITY

**FIGURE 2:**
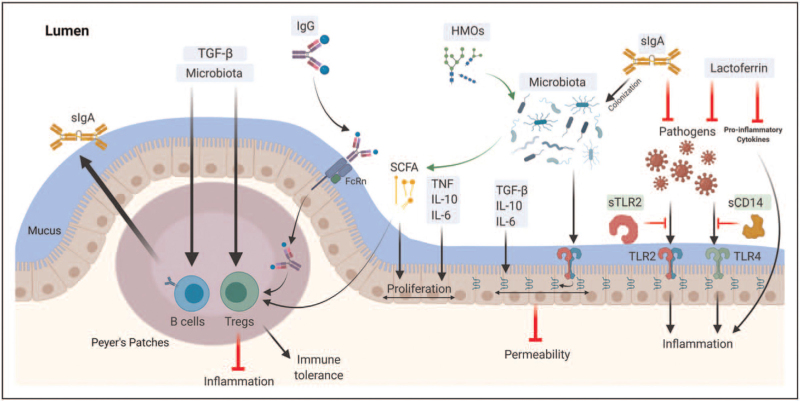
Milk immune factors and mucosal immunity. (Original). Several aspects of the mucosal immune system are developed under the influence of human milk components in early life. Maternal sIgA is the most abundant immunoglobulin in human milk; it inhibits pathogens and facilitates colonization of commensal microbiota. Maternal IgG is present at lower levels in milk and can be found complexed with dietary antigens; these complexes can translocate across the gut epithelium through the neonatal FcRn receptor and then stimulate expansion of regulatory T cells (Tregs). Human milk cytokines, including the TGF-β family, IL-6, IL-10, and TNF, are essential in the homeostasis of mucosal immunity via enhancing epithelial integrity and proliferation, expanding Tregs and endogenous sIgA secretion from infant B cells. Lactoferrin blocks pro-inflammatory cytokines and free-radical activity, and inhibits pathogen growth by chelating iron. Human milk microbiota may colonize the infant's gut and promote the development of mucosal immunity, including intestinal integrity, Tregs expansion, and sIgA production, either directly via signaling through innate immune receptors or indirectly via metabolizing human milk oligosaccharides (HMOs) into short-chain fatty acids (SCFAs) that serve as an energy source for gut epithelial cells. Soluble receptors in milk (such as sTLR2 and sCD14) are essential in regulating the inflammatory response, acting as decoy receptors that bind pathogenic bacterial ligands.

The World Health Organization recommends 6 months of exclusive breastfeeding, with continued breastfeeding together with appropriate complementary foods up to 2 years of age or longer [38]. A primary reason for this recommendation is that breastfeeding fills a critical gap in neonatal/infant immunity by transferring bioactive factors that provide passive immune protection and stimulate normal mucosal immune development, supporting lifelong homeostasis (Table 1 and Fig. 2).

**Table 1:** Human milk components with immunomodulating activity

Component	Immunomodulating activity	References
**Immunoglobulins**		
sIgA (∼85% of total Igs in milk)	• Neutralize pathogens while supporting commensal bacteria• Regulate inflammatory responses to microbial antigens• Promote intestinal homeostasis & mucosal immune tolerance	[114,115]
IgGs	• Bind specific antigens (including allergenic foods) & transport them across the gut barrier through binding to intestinal FcRn, promoting tolerance	[44,46,47]
IgM	• Strong complement-fixing activity	[116]
IgE	• Binds to mast cells and basophils may benefit antiparasitic responses	[117]
**Cytokines**		
TGF-β1, TGFβ-2	• Inhibit T cell differentiation• Induce Treg expansion• Promote intestinal integrity• Stimulate B cell class switching to IgA• Promote microbial colonization• Promote mucosal tolerance	[73–79]
IL-4, IL-5, IL-13	• Promote Type 2 cytokine responses and T cell differentiation	[118]
IL-10	• Multiple anti-inflammatory roles and mast cell growth factor	[119]
TNF, IL-6, IFN-γ, IL-12	• Drivers of inflammatory responses and Type 1 responses	[120]
GMCSF, GCSF, MCSF	• Granulocyte, macrophage and dendritic cell growth and differentiation factors	[121]
**Chemokines**		
CCL4, CCL5, CXCL10	• Mediate immune and stromal cell recruitment to sites of infection, damage or inflammation	[122]
**Soluble Receptors**		
Innate receptors: sTLR2, sCD14	• Bind microbial ligands, attenuate inflammatory response	[90,123,124]
Cytokine receptors: IL-1RA, sIL-6R, sTNF-RI, sTNF-RII	• Regulate signaling of milk-borne IL-1b, IL-6, TNF	[86,87]
Other		
Epithelial Growth Factors: IGF-I, EGF	• Promote epithelial development and repair	[125]
Maternal cells (∼80% myeloid cells, 20% lymphocytes)	• Migrate into gut mucosa and/or pass into systemic circulation• Contribute to antibody responses• Regulate T cell development and responses	^56^ ^57^ ^1^ ^2^ ^3^ ^4^ ^5^ ^6^ ^7^ ^8^ ^9^ ^10^ ^11^ ^12^ ^13^ ^14^ ^15^ ^16^ ^17^ ^18^ ^19^ ^20^ ^21^ ^22^ ^23^ ^24^ ^25^ ^26^ ^27^ ^28^ ^29^ ^30^ ^31^ ^32^ ^33^ ^34^ ^35^ ^36^ ^37^ ^38^ ^39^ ^40^ ^41^ ^42^ ^43^ ^44^ ^45^ ^46^ ^47^ ^48^ ^49^ ^50^ ^51^ ^52^ ^53^ ^54^ ^55^ ^56^ ^57^ ^58^ ^59^ ^60^
Microbiota	• Some may seed the infant gut• Compete with pathogens for ecological niche• Produce SCFAs that support epithelial proliferation & improve gut barrier• Enhance gut barrier integrity by binding innate immune receptors (e.g. TLR2) on gut epithelium	[18^▪^,^93^,^98^,^99^]
Oligosaccharides	• Prebiotic substrate for gut microbiota• Antiadhesive properties block host-pathogen interactions• Decoy receptor for some pathogens• Co-factor for some viral binding• Absorption into circulation: systemic activities	[104–106,126]
Lactoferrin	• Chelate iron• Block pro-inflammatory cytokines & free radical activity• Bacteriostatic function against pathogens requiring iron• Support selective microbiota growth, including *Bifidobacteria* and *Lactobacilli*	[4^▪^,^109^–^112^]

CD, cluster of differentiation; EGF, epidermal growth factor; FcRn, neonatal Fc receptor; GCSF, granulocyte colony-stimulating factor; GMCSF, granulocyte-macrophage colony-stimulating factor; IFN-γ, interferon-gamma; Ig, immunoglobulin; IGF-I, insulin-like growth factor-1; IL, interleukine; MCSF, macrophage colony-stimulating factor; TGF, transforming growth factor; TLR, toll-like receptor; TNF, tumor necrosis factor.

### Immunoglobulins

Human milk immunoglobulins, including IgA, IgGs, IgM, IgE, and IgD, are critical in modulating and shaping neonatal mucosal immunity [39^▪^,^40^^▪▪^]. Human milk is the only source of sIgA during the first month of life. sIgA comprises around 80–90% of total immunoglobulins in milk [4^▪^], with its highest concentration in colostrum [41]. sIgA is the first line of antigen-specific defense that can effectively neutralize pathogens while permitting commensal bacteria [4^▪^], suggesting a vital role in microbiota colonization and intestinal homeostasis. In addition, the majority of intestinal bacteria are coated with sIgA, which helps regulate inflammatory responses to microbial antigens [42]. Furthermore, allergic disease incidence among breastfed infants has been negatively associated with levels of sIgA in their mothers’ milk [43], indicating an essential role in mucosal immune tolerance.

IgG subtypes are also detected in human milk at much lower levels than sIgA. They can bind specific antigens, fix complement and enhance uptake of antigens. Food antigens are found complexed with IgG in maternal serum and are transferrable into breastmilk via the Fc receptor (FcRn) expressed in the mammary gland's epithelium [44]. FcRn is also found in the neonatal intestine where it mediates the translocation of the IgG-allergen complex across the gut barrier [45] where they can be taken up by FcRn-bearing antigen-presenting cells. Allergens bound to IgG have been shown to induce more Tregs and more profound immune tolerance than free allergens [46], suggesting that exposure to IgG-complexed food antigens through breastmilk may contribute to food allergy prevention [47]. This hypothesis is supported by recent data from the CHILD Cohort Study, where infants who were introduced to peanut before weaning and breastfed by mothers who regularly consumed peanuts had a reduced risk of peanut sensitization and allergy through 5 years of age [48^▪^].

Interest in human milk antibodies has surged during the Covid-19 pandemic, with multiple groups [49^▪^,^50^,^51^^▪▪^,^52^,^53^] showing that mothers who recover from Covid-19 have antigen-specific sIgA in their breastmilk that is presumed to protect their infants. Vaccination-induced antibodies are also present in human milk, although the profile is different (IgG-dominant) than following natural infection (IgA-dominant) [54^▪▪^,^55^].

### Immune cells

Despite the challenging environment of the infant digestive system, the transfer of maternal cells continues after birth through breastmilk. As recently reviewed [56^▪^], these cells can migrate into the gut mucosa and may even pass into the systemic circulation where they can contribute to neonatal antibody responses and regulation of T cell responses, including their repertoire [57–60]. Myeloid cells such as monocytes, macrophages and DCs are predominant in breastmilk, making up more than 80% of total leukocytes [56^▪^]. Lymphocytes make up the bulk of the remaining 20%; these are predominately T cells but also include natural killer cells and subsets of B cells. The composition and function of these cells vary substantially during lactation and in response to other factors [61]. Various roles have been attributed to these maternal immune cells in milk, including microchimerism-induced immune maturation [62] and the enhancement of allergy development through IgE transport [63]. An important recent finding is the observation that, in mice, the transfer of IgA and IgA-producing cells through milk can impact the development of regulatory T cells in the offspring. These elegant cross-fostering experiments identify a novel mechanism by which immune function can be transmitted across generations independently of genetic, epigenetic or microbial factors [64^▪▪^].

### Cytokines, growth factors, and soluble receptors

Cytokines and growth factors in milk are signaling proteins involved in cell communication, recruitment, regulation and stimulation. A wide array of these immune factors are found in breastmilk, including TGF-β1, TGF-β2, IL-10, IL-6, IL-1β, tumor necrosis factor (TNF), insulin-like growth factor (IGF)-1, IFN-γ, IL-4, IL-5, IL-12, IL-13, G-colony-stimulating factor (CSF), GM-CSF, and M-CSF [43,65–69], and the list is expanding. These immune mediators tend to be present at higher concentrations in colostrum than mature milk, indicating their potential importance in supporting immature mucosal immunity in neonates.

The most abundant cytokines in human milk, TGF-β2 and TGF-β1 [43], are scarce in infant formula [70–72]. The role of the TGF-β family in mucosal immunity is multifactorial, including inhibition of T cell differentiation into effector Th1 and Th2 cells [73,74], inducing Tregs’ expansion [75,76], promoting intestinal integrity [77], stimulating B cell class switching to IgA [78], and promoting microbial colonization [79]. According to Järvinen *et al.,*[80,81], higher levels of milk TGF-β1, along with IL-1β, IL-10, and IL-6, associate with a decrease in food allergy, suggesting a role of these cytokines in mucosal tolerance. Animal studies have also shown that IL-10 [82], IL-6 [83], and TNF [84] impact intestinal cell maturation and proliferation. Notably, levels of cytokines in milk and their impact on immune development are highly dependent on maternal genetics and immune system status. For example, mouse pups nursed by dams deficient in the pattern recognition receptor TLR2 have attenuated epithelial barrier function and altered responses to oral antigens [85^▪^].

Breastmilk also contains bioactive soluble receptors that can regulate cytokine and bacterial signaling by sequestering them away from the neonatal mucosa. For example, cytokine receptors and antagonists, including IL-1RA, sIL-6R, sTNF-RI, and sTNF-RI, are detected in human milk and are believed to regulate the signaling of milk-borne IL-1β [86–88], IL-6 [89], and TNF [86] cytokines, respectively. Soluble forms of innate immune receptors are also detected in human milk, including sTLR2 and sCD14 [90]. These soluble receptors bind to different microbial ligands leading to attenuation of the inflammatory response in infants.

### Microbiota

It is now accepted that breastmilk is not sterile [91]. Our group and others have described a diverse human milk microbiome that, like other milk components, appears to be dynamic and highly variable among mothers [91,92]. Milk-borne microbiota may contribute to the developing infant gut microbiota, which promote mucosal development by competing with pathogens for the ecological niche, producing bioactive factors that improve the intestinal barrier, and educating the mucosal immune system [18^▪^,^93^,^94^]. Recent data from the CHILD Cohort Study identified specific microbial taxa that are commonly shared between a mother's milk and her infant's gut microbiota and showed that this 'sharing of bacteria’ is increased among exclusively breastfeeding dyads [95^▪^]. Certain commensal microbes, including *Bifidobacteria* and *Lactobacilli*, are enriched in the gut of breastfed (vs. formula fed) infants and have been shown to enhance intestinal barrier integrity [96,97] through binding innate immune receptors (e.g., TLR2) on the gut epithelium [98]. In addition, the digestion of human milk oligosaccharides (HMOs, not found in most infant formulas) by *Bifidobacterium* and *Lactobacillus* species in the neonatal intestine produces short-chain fatty acid (SCFA) including butyrate, acetate, and propionate [99]. These SCFAs enhance epithelial proliferation, gut barrier function, and intestinal motility [100]; they also serve as an energy source for host enterocytes, and have been associated with protection from allergic diseases [101].

### Oligosaccharides

Oligosaccharides are complex glycans present in human milk in higher concentrations than any other mammalian milk, with over 150 structurally distinct HMOs identified thus far [102^▪^]. Each mother secretes a unique HMO profile [103]. HMOs serve a prebiotic function in human milk, acting as substrates for the neonatal gut microbiota [104]. Some HMOs can also act as decoy receptors that block microbial binding to epithelial cells by mimicking the epithelial cell receptors [105]. In addition, some HMOs can be absorbed into the circulation and interact with immune and epithelial cells, thus, modulate the neonatal immune response [106]. New *in vitro* data from Cheng *et al.* shows that HMOs may also directly enhance mucosal barrier function by modulating intestinal goblet cell gene expression [107^▪^]. In addition, recent experiments in human and mouse model systems have shown that the HMO disialyllacto-N-tetraose favorably modulates mast cell homeostasis, contributing to protection against necrotizing enterocolitis, an often fatal gastrointestinal disorder in preterm neonates [108^▪▪^].

### Lactoferrin

Lactoferrin is an iron-binding glycoprotein that is highly abundant in human milk, particularly in colostrum [109]. It has a bacteriostatic function via preventing the growth of iron-dependent pathogens [110]. Lactoferrin also inhibits the binding of pathogens to host cells and regulates excess immune responses via blocking pro-inflammatory cytokines and free-radical activity [4^▪^,^111^,^112^]. At the same time, lactoferrin has been shown to regulate intestinal homeostasis [4^▪^] and support selective microbiota growth, including *Bifidobacteria* and *Lactobacilli*[113].

## CONCLUSION

During infancy, the mucosal immune system is ’programmed’ through exposure to environmental, dietary, and microbial factors, with potentially life-long implications. Maternal milk is the primary source of nutrients and immune factors during this critical period of development. The beneficial impact of breastfeeding on mucosal immune development is supported by the decrease in morbidity and mortality among optimally breastfed infants. However, more research is required to understand how human milk components interact with the neonatal mucosa to support immune development. This knowledge will help inform disease prevention and treatment strategies for infants (including those who cannot be breastfed), and could also inform therapeutic approaches to immune-mediated conditions in older children and adults.

## Acknowledgements

*We thank Rilwan Azeez for his critical and constructive review of this article.*

### Financial support and sponsorship

*This work was supported by a Canadian Institutes of Health Research Project Grant #156155 and a Bill & Melinda Gates Foundation Grant #INV-001734. M.B.A. is supported by Tier 2 Canada Research Chair in the Developmental Origins of Chronic Disease at the University of Manitoba and is a Fellow in the CIFAR Humans and the Microbiome Program.*

### Conflicts of interest

*M.B.A. regularly speaks at conferences and workshops on infant nutrition. She has received speaking honoraria from Prolacta Biosciences and AstraZeneca. She has contributed without remuneration to online courses on breastmilk and the infant microbiome produced by Microbiome Courses. She serves in a volunteer capacity for the International Society for Research on Human Milk and Lactation and as a member of the National Academy of Sciences, Engineering and Medicine Committee on Scanning New Evidence on the Nutrient Content of Human Milk. She also serves on the Malaika Vx Scientific Advisory Board. B.D. and J.S.M. declare no conflicts of interest.*

## REFERENCES AND RECOMMENDED READING

Papers of particular interest, published within the annual period of review, have been highlighted as:

▪ of special interest
▪▪ of outstanding interest
